# Regional difference in the rate of spread of SARS-CoV-2

**DOI:** 10.1017/ice.2020.223

**Published:** 2020-05-11

**Authors:** Kazuhiro Tanabe, Katsuhiko Sasaki, Ko Igami, Kazuyuki Kamioka

**Affiliations:** 1 Medical Solution Segment, LSI Medience, Tokyo, Japan; 2 Kyushu Pro Search, Japan; 3 Business Management Division, Clinical Laboratory Business Segment, LSI Medience, Tokyo, Japan

*To the Editor*—After the first case of coronavirus disease 2019 (COVID-19), caused by severe acute respiratory syndrome coronavirus 2 (SARS-CoV-2), was reported in Wuhan, China, in December 2019, the total number of confirmed cases had risen to 1,800,000, globally, and the total number of deaths had exceeded 120,000 by April 15, 2020.^1^ During this period, vast epidemiological data were collected globally that can provide clues about how to address this pandemic.

Here, we focus on the regional differences in the rate of spread of SARS-CoV-2 in Asia, Europe, and North America, and we discuss how these differences arose. All of the data we analyzed were obtained from the World Health Organization.^1^

**Fig. 1.**
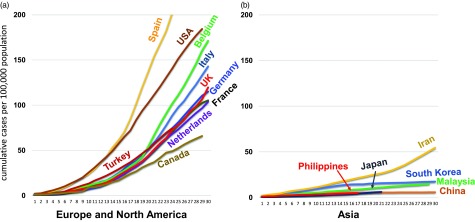
Cumulative incidence of SARS-CoV-2 infection in countries in Europe, North America, and Asia.

To assess the regional differences in the rate of spread, we prepared growth curves of the cumulative cases per 100,000 population in 16 countries by calculating the ratio between the total number of confirmed cases and the country’s population. The target countries were chosen using 2 criteria: countries (1) with >10 million population and (2) with >3,000 confirmed cases by April 6, 2020. Overall, 6 Asian countries met these criteria (China, Japan, South Korea, Malaysia, Philippines, and Iran); 8 European countries met these criteria (France, Germany, Spain, Italy, United Kingdom, Belgium, Netherlands, and Turkey); and 2 North American countries met these criteria (United States and Canada). The growth curves for the subsequent 30 days were plotted, starting once the cumulative incidence exceeded 1 case per 100,000 population. India was not included, despite meeting the first 2 criteria because it had not reached the cumulative incidence threshold of 1 case per 100,000 population by April 6, 2020. This analysis revealed that the cumulative incidence of European and North American countries rose exponentially in a similar manner (Fig. 1a), whereas those of Asian countries did not (Fig. 1b).

The rate of spread is shown by the slopes of the cumulative incidence curves in (1) Europe and North America and (b) Asia. The cumulative incidence is shown per 100,000 population.

A Student *t* test of the difference in cumulative incidence per 100,000 population at 17 days in Asian countries compared to that in European and North America countries revealed a statistically significant difference (*P* < 0.001), suggesting that some underlying factors may be affecting the rate of spread.

We questioned why the rate of spread of SARS-CoV-2 has been much slower in Asian countries than in European and North American countries. The reason for the small number of confirmed cases and total deaths due to SARS-COV-2 in Japan has been a subject of debate. Some have attributed it to the high level of discipline exhibited by Japanese people, such as full-time wearing of face masks and frequent handwashing. However, we propose that the limited number of PCR tests conducted is the main reason for the small number of confirmed cases and that the underdeveloped private practice system and strict government policy have prevented people from undergoing PCR testing in Japan.^2^ Although the low cumulative incidence per 100,000 population in Japan is striking, the incidence rates of the other Asian countries are also much lower than those of European and North American countries.

The small number of cases reported by China and South Korea since the incidence peaked in these countries has also been a subject of debate. Despite the large size of the susceptible population in China and South Korea, the number of new cases per day has been 100 or less since the middle of March 2020. One hypothesis is that Bacillus Calmette-Guérin (BCG) vaccination protects against respiratory viruses.^3^ BCG vaccination is routine in most Asian countries; however, scientific evidence supporting that BCG protects against coronavirus is currently lacking. Habitual physical contact (hugging, kissing, and shaking hands) or hesitation in wearing face masks in public have been proposed as reasons for the faster spread in Europe and North America.

These hypotheses may account for some of the differences in the of spread among regions; however, we present a new hypothesis that Asians have resistance to coronaviruses. There have been several coronavirus outbreaks in Asia in the past. SARS coronavirus caused an outbreak in China in 2002 (8,422 persons were infected and 916 people died in 37 countries)^4^; Middle East respiratory syndrome (MERS) coronavirus led to 858 deaths due to severe respiratory illness in Saudi Arabia in 2012^5^ and in South Korea in 2015.^6^

Considering the multiple opportunities for exposure to coronavirus in Asia, Asian people may have acquired some degree of immunological or genetic resistance to coronaviruses through repeated exposure over a long period, and this might have reduced the impact of the COVID-19 pandemic in Asia. We acknowledge the need for further research to determine the reasons for the variable rate of spread; however, the COVID-19 pandemic could provide clues that could help prevent future pandemics.
